# HealthSWEDE: costs with sublingual immunotherapy—a Swedish questionnaire study

**DOI:** 10.1186/s13223-021-00560-3

**Published:** 2021-06-07

**Authors:** Petter Olsson, Carl Skröder, Lars Ahlbeck, Frida Hjalte, Karl-Olof Welin, Ulla Westin, Morgan Andersson, Cecilia Ahlström-Emanuelsson, Lars-Olaf Cardell

**Affiliations:** 1 Division of Ear, Nose and Throat Diseases, Department of Clinical Sciences, Intervention and Technology, Klinisk Vetenskap (CLINTEC), Karolinska Institutet, Intervention och teknik, H9 Öron-, näs, och halssjukdomar, 171 77 Stockholm, Sweden; 2 Department of Otorhinolaryngology, Head and Neck Surgery, Institute of Clinical Sciences, Skåne University Hospital, Lund, Sweden; 3 Allergy Center, Linköping University Hospital, Linköping, Sweden; 4 The Swedish Institute for Health Economics (IHE), Lund, Sweden

**Keywords:** Absenteeism, Direct costs, Presenteeism, Allergic rhinitis, Indirect costs

## Abstract

**Background:**

The aim of this cross-sectional survey was to compare the health-economic consequences for allergic rhinitis (AR) patients treated with sublingual Immunotherapy (SLIT) in terms of direct and indirect costs with a reference population of patients receiving standard of care pharmacological therapy.

**Methods:**

Primary objective was to analyse the health-economic consequences of SLIT for grass pollen allergy in Sweden vs reference group waiting for subcutaneous immunotherapy (SCIT). A questionnaire was mailed to two groups of AR patients.

**Results:**

The questionnaire was distributed to 548 patients, 307 with SLIT and 241 in reference group (waiting for SCIT). Response rate was 53.8%. Mean annual costs were higher for reference patients than SLIT group; € 3907 (SD 4268) vs € 2084 (SD 1623) p < 0.001. Mean annual direct cost was higher for SLIT-patients, € 1191 (SD 465) than for reference, € 751 (SD 589) p < 0.001. Mean annual indirect costs for combined absenteeism and presenteeism were lower for patients treated with SLIT, € 912 (SD 1530), than for reference, € 3346 (SD 4120) p < 0.001, with presenteeism as main driver.

**Conclusions:**

SLIT seems to be a cost-beneficial way to treat seasonal AR. This information might be used to guide future recommendations.

**Supplementary Information:**

The online version contains supplementary material available at 10.1186/s13223-021-00560-3.

## Background

Allergic rhinitis (AR) is a global health problem with adverse impact on sleep, cognitive function, mood, and comorbid conditions, such as asthma [1]. The prevalence in Sweden is approximately 30% with birch and grass pollen as the dominating allergens [2]. AR causes, on top of the individual burden, additional costs at a societal level, particularly in terms of increased healthcare utilization, reduced productivity and impairment activities of daily living. Effects on society, in terms of work absence (absenteeism), impaired work productivity while at work (presenteeism), as well as loss of school days, have recently been acknowledged as an issue [3], even though the scientific knowledge in the field is still scarce. (Additional file 1: Table S1)

Intranasal corticosteroids and oral and/or ocular antihistamines constitute the foundation of AR treatment, sometimes with the addition of systemic steroids when seasonal symptom control fails. However, allergen immunotherapy (AIT) is the only therapy that, besides alleviating symptoms, also appears to improve the long-term development of the disease [4]. Subcutaneous immunotherapy (SCIT) has been used for decades, whereas sublingual immunotherapy (SLIT) has been available for about 10 years. Both therapies have been proven efficacious in the treatment of pollen induced symptoms, but their use is limited by drawbacks. SCIT requires, besides an induction period of about three months with weekly physician supervised subcutaneous injections, continued every 6–8 weeks during 3–4 years. SLIT is based on a daily tablet intake during the same period. It has been considered to be a relatively expensive treatment due to the cost of the medication. Previous studies have indicated that AIT might ease the socioeconomic burden caused by AR [5]. Hence, the aim of the present cross-sectional survey, “HealthSWEDE” or Health economy and sublingual immunotherapy in Sweden, was to compare the health-economic consequences for patients treated with SLIT, in terms of direct and indirect costs with patients receiving standard of care pharmacological therapy. (Additional file 2)

## Methods

### Study design and population

This study was a cross-sectional survey including patients 18–65 years old identified via specialist centres in Sweden. Two groups of patients with specific IgE-confirmed grass pollen allergy were identified; one had received at least 12 months of SLIT treatment against grass pollen allergy, n = 307, whereas the other cohort of individuals, the reference population, were waiting to start up SCIT against grass pollen and/or birch pollen allergy, n = 241. During the waiting period the latter group had received standard of care pharmacological treatment. The definition of “standard of care” was, per protocol, left to the judgement of the investigators, which were all experienced physicians (ENTs or Pulmonologists or Allergy specialists) working at specialized allergy or ENT-centers in Sweden that perform allergen immunotherapy on a routine basis.

The optimal reference group for the SLIT cohort would have been patients waiting for SLIT, but since SLIT in Sweden usually starts more or less upon referral, there is practically no waiting list for this therapy. Thus, patients waiting for SCIT were used for the comparison. Only Grazax^®^, also known as Grastek^®^, 75,000 SQ-T tablets (ALK-Abelló A/S, Denmark) is available for grass pollen SLIT in Sweden.

A questionnaire was mailed, after the birch and grass seasons, to the two groups of AR patients with a valid postal address in Sweden. Distribution was done by regular mail in September 2017 (a postal reminder was sent out approximately 2 weeks after the first letter) to capture the seasonal allergies season from March to August the same year. The questionnaire included questions on age, gender, employment status, sick leave, health care resource utilization during the past year and quality of life as measured by EQ-5D-3L [6].

Inclusion criteria were age 18–65 years, a valid address in Sweden and prick test or specific IgE-confirmed grass pollen allergy. The only exclusion criterion was lack of ability to read and write Swedish.

The primary objective of this cross-sectional survey was to analyse the health-economic consequences in terms of direct and indirect costs and quality of life in the treatment of grass allergy with SLIT for grass pollen allergy in Sweden vs a reference group with standard of care, waiting for SCIT.

### Health-economic analyses

Direct costs were calculated for pharmaceuticals and for health care contacts related to allergic nasal/eye problems (physician visits, nurse visits and telephone consultations). Unit costs for health care contacts were collected from the price list for the Southern Health Care Region [7]. Unit costs for pharmaceuticals were collected from “FASS” (Pharmaceutical Specialities in Sweden, the Swedish Drug Information site) [8].

When calculating the number of days for questions answered with intervals, the middle value for the interval was used. In cases where a response gives more than a certain value, the minimum exceeding the value was used to provide a conservative estimate. Costs were not calculated for SCIT treatment in accordance with the definition of the included population.

Indirect costs included costs for productivity loss due to both sick-leave, i.e. absenteeism, and impaired working capacity, i.e. presenteeism. Productivity losses were calculated according to the human capital approach [9] using the average yearly income from work and adjusting for payroll taxes by multiplying by 1.43 (the mean employment payroll taxes for the Swedish population is 43%) [10–12]. The maximum occupation level was assumed to be 100%. For respondents reporting several part-time occupations the sum of the part-time occupations was used up to 100%. For respondents reporting a part-time occupation without specifying the extent, a 50% occupation level was assumed.

The productivity loss due to absenteeism was calculated by multiplying days of reported sick leave by sex and age-adjusted mean wage including payroll taxes. Productivity loss due to presenteeism was likewise calculated but multiplied with the estimate where the respondent indicated to what extent productivity was reduced while working with allergic nasal eye problems. When calculating costs related to presenteeism and absenteeism, answers indicating a longer season than 4 months are set to only include the four months relevant to the pollen season as not to overestimate the indirect costs.

The presented mean costs were calculated on a sample limited to the working population, i.e. approximately 80% of the total study population. This approach was used to avoid skewing the mean total and indirect costs (Additional file 1: Table S1).

Swedish costs were converted to Euros, €, using the average exchange rate 2017.

### Statistical analyses

Descriptive statistical analyses were conducted for all questions in the survey and for direct, indirect and total costs. Differences between groups were assessed with t-tests for continuous variables and Chi-squared tests were used for categorical variables. All tests were performed at the 0.05 level of significance and were two-sided.

Statistical analyses were performed using STATA, version 14 for Windows (StataCorp, Stata Statistical Software: Release 14. 2015, StataCorp LP: College Station, TX, USA).

## Results

### Sample characteristics

**Fig. 1:**
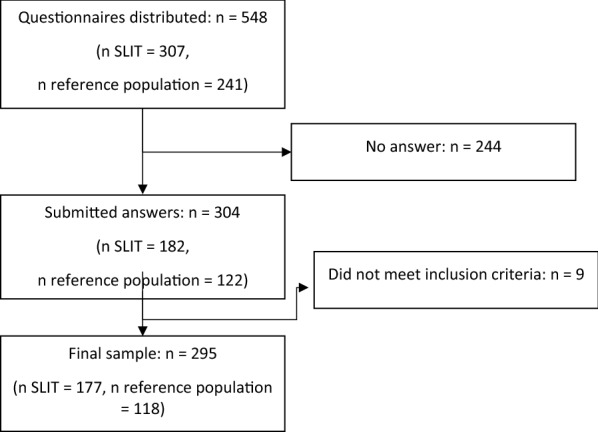
Flow chart of study population

The questionnaire was distributed to 548 patients, 307 treated with SLIT and 241 in the reference group, waiting for SCIT. The questionnaire was answered by 304 patients out of which 295 fulfilled inclusion criteria, corresponding to a total response rate of 53.8% (shown in Fig. 1). For details on patient characteristics, please see Table 1.

**Table 1:** Patient characteristics for patients with grass pollen allergy with and without sublingual immunotherapy (SLIT)

	Total (n = 295)	SLIT (n = 177)	Reference population (n = 118)
Men/women	49%/51%	55%/45%	41%/59%
Age, mean years (SD)	36.5 (11.1)	37.2 (11.6)	35.3 (10.2)
Employed, n (%)	235 (80%)	146 (82%)	89 (75%)
Daily smoker, n (%)	6 (2%)	5 (3%)	1 (< 1%)
Previous smoker, n (%)	52 (18%)	37 (21%)	15 (13%)
Self-reported asthma, n (%)	97 (33%)	57 (32%)	40 (34%)
Self-reported eczema, n (%)	53 (18%)	30 (17%)	23 (19%)
Self-reported use of medications against nasal and or eye problem during the last year, n (%)	282 (96%)	168 (95%)	114 (97%)
Out of those^a^			
Systemic corticosteroids, n (%)	69 (24%)	24 (14%)	45(41%)
Nasal steroid spray, n (%)	224 (79%)	122 (73%)	102 (89%)
Nasal antihistamine spray, n (%)	92 (33%)	41 (24%)	51 (45%)
Antihistamine, oral, n (%)	245 (87%)	138 (82%)	107 (94%)

^a^Total (n = 282), SLIT (n = 168) Reference population (n = 114)

### Costs

**Fig. 2:**
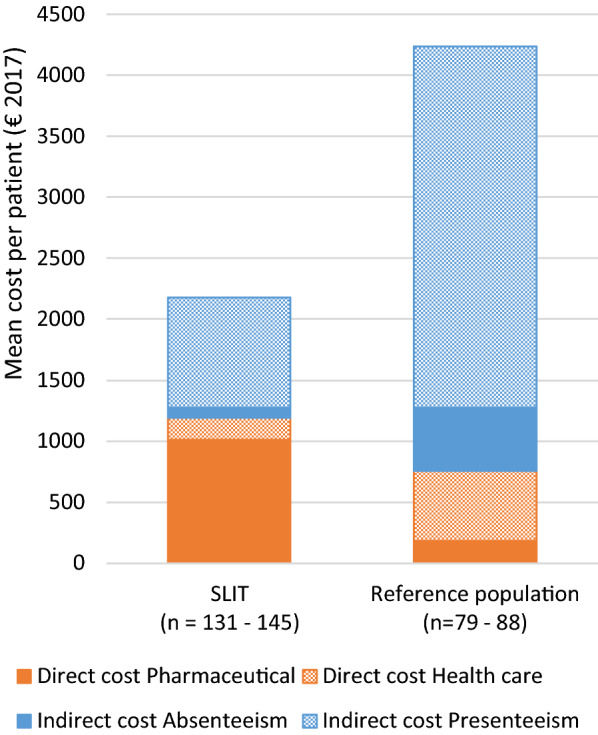
Direct (pharmaceutical or health care) and indirect costs (absenteeism or presenteeism) by treatment group; € 2 084 (SD 1623) for the SLIT group vs € 3 907 (SD 4268) for reference population (p < 0.001). SLIT, Sublingual Immunotherapy. Reference population, Standard of care. Mean cost per patient/year

The mean annual pharmaceutical costs were higher among SLIT patients than among the population controls, € 1014 (Standard Deviation, SD 325) vs € 183 (SD 123, p < 0.001). The reference population patients on the waiting lists had, according to their treating specialist, been subject of standard of care pharmacological treatment optimization. In spite of this, the SLIT group only had mean annual health care costs of € 179 (SD 347) vs € 574 (SD 538, p < 0.001) for the reference patients on the waiting list. The total mean annual direct cost was significantly higher for patients with SLIT, € 1191 (SD 465) than for reference patients, € 751 (SD 581, p < 0.001). The total annual indirect costs for the combined absenteeism and presenteeism were markedly lower for patients treated with SLIT, € 912 (SD 1530), than for reference, € 3346 (SD 4120, p < 0.001), with presenteeism as the main driver (shown in Fig. 2). Altogether, the mean annual total costs were significantly higher for reference patients not treated with SLIT than for the SLIT group; € 3907 (SD 4268) vs. € 2084 (SD 1623), respectively, p < 0.001. A numerical improvement of quality of life, as measured by EQ-5D-3L, was reported by the patients with SLIT (mean 0.889) compared to the reference population (mean 0.847, p = 0.072).

## Discussion

In this study, sublingual immunotherapy was more cost beneficial compared to standard of care pharmacological treatment of seasonal allergic rhinitis, mainly due to reduced indirect costs (absenteeism and presenteeism).

US population-based surveys have estimated the annual number of workdays missed due to AR to be in the range of 0.03–0.8 per employed individual per year [13]. Goetzel et al. have, by combining data on work productivity impairment from three large-scale US surveys, concluded that allergy, excluding asthma, was associated with an average 3% loss of productivity due to work absence and an average 11% reduction in at-work performance [14]. A Spanish study found statistically significant associations between loss of work and academic productivity, impairment of daily activities and the type and severity of AR. AIT was a protective factor [15]. We have previously investigated the health economic burden attributed to all rhinitis in general as well as AR specifically in the Swedish population. The total annual cost for the former was € 2.7 billion and the later € 1.3 billion [16, 17].

The high prevalence of AR makes it clear that even a limited improvement of the therapeutic outcome would significantly ease the socioeconomic burden of this disease on the society. It also important to notice that the present calculation of the economic savings with SLIT is made during the treatment period and that according to previous studies the effect of the therapy will remain far beyond this period thereby further increasing the gains to society. On the other hand, a recent study claims that AIT for grass pollens may be a cost-effective option only in patients with low discontinuation rates and that SCIT, which is less affected by this limitation than SLIT, could be the most cost-effective AIT form [18]. A cost-minimization analysis from Canada indicates that house dust mite SLIT-tablets are a cost-minimizing alternative to HDM SCIT in treating allergic rhinitis when considered from a societal perspective in the Ontario and Quebec provinces [19].

A strength of this study includes the fact that even though an increasing number of studies of AR have included quantitative and validated measures of absenteeism and presenteeism this is, to our knowledge, the first cross-sectional survey that assesses the total, direct and indirect annual costs of SLIT in the treatment of seasonal allergic rhinitis. The fact that this study captured indirect costs due to presenteeism is important, especially as this seems to be a significant part of total costs in individuals with allergic rhinitis.

Some limitations in the method we had to use is that the two comparison groups were not exactly equivalent in some characteristics. Despite being a short and not very time-consuming instrument, EQ-5D could be considered less sensitive to improvements in health related quality of life than a longer disease-specific measure, such as the Rhinoconjunctivitis Quality of Life Questionnaire. However, we chose the EQ-5D to increase probability for a high response rate. Furthermore, the retrospective design, i.e. respondents reported allergic symptoms, health-care resource utilization and quality of life for a period back in time, could imply a recall bias which recommends caution when interpreting the results. Although very similar questionnaires investigating allergic rhinitis in the Swedish population have been used previously by our group [16, 17], the questionnaire, excluding EQ-5D-3L, has not formally been exposed to a full validation process.

## Conclusion

SLIT seems to be a cost-beneficial way to treat seasonal allergic rhinitis, and this information might be used to guide both future recommendations for clinical practice and public health interventions.

## Supplementary Information

**Additional file 1: Table S1.** Total cost for patients ( n =295) with grass pollen allergy with and without sublingual immunotherapy (SLIT), € 2017.

**Additional file 2.** Questionnaire.

## Data Availability

The datasets used and analyzed during the current study are available from the corresponding author on reasonable request.

## Abbreviations

AR: Allergic rhinitis
SLIT: Sublingual immunotherapy
SCIT: Subcutaneous immunotherapy
AIT: Allergen immunotherapy
IgE: Immunoglobulin E
FASS: “Farmaceutiska Specialiteter i Sverige” (Pharmaceutical specialties in Sweden)
SD: Standard deviation
€: Euros
