# Supplementary material for: HealthSWEDE: costs with sublingual immunotherapy—a Swedish questionnaire study
Source: Allergy Asthma Clin Immunol. 2021 Jun 7;17:55. doi: 10.1186/s13223-021-00560-3 (PMC8183062; doi:10.1186/s13223-021-00560-3)
Supplement: Supplementary file 1 — Additional file 1: Table S1. Total cost for patients ( n =295) with grass pollen allergy with and without sublingual immunotherapy (SLIT), € 2017. [file 13223_2021_560_MOESM1_ESM.docx]

**Additional file 1**

**HealthSWEDE: Costs with sublingual immunotherapy - a Swedish questionnaire study**

**Authors**

Petter Olsson, Carl Skröder, Lars Ahlbeck, Frida Hjalte, Karl-Olof Welin, Ulla Westin, Morgan Andersson, Cecilia Ahlström Emanuelsson, Lars-Olaf Cardell

**Table S1**

|  | **Total** | **SLIT** | **Reference population** | **p-value** |
| --- | --- | --- | --- | --- |
| ***Direct cost*** | *(n=295)* | *(n=177)* | *(n=118)* |  |
| *Mean (SD)* | 1036 (561) | 1210 (432) | 775 (629) | < 0.001 |
| *Median (IQR)* | 1136 (657-1284) | 1179 (1120-1346) | 678 (346-929) |  |
| ***Indirect cost*** | *(n=225)* | *(n=142)* | *(n=83)* |  |
| *Mean (SD)* | 1810 (3012) | 912 (1530) | 3346 (4120) | < 0.001 |
| *Median (IQR)* | 789 (71-2143) | 330 (0-1250) | 2078 (592-4149) |  |
| ***TOTAL COST*** | *(n=295)* | *(n=177)* | *(n=118)* |  |
| *Mean (SD)* | 2417 (2802) | 1942 (1502) | 3129 (3924) | < 0.001 |
| *Median (IQR)* | 1483 (1138-2624) | 1409 (1173-2275) | 1735 (758-3950) |  |

SLIT, Sublingual Immunotherapy. SD, standard deviation. IQR, interquartile range.

**Legend for Table S1**

Total cost for patients (n=295) with grass pollen allergy with and without sublingual immunotherapy (SLIT), € 2017.
