# Supplementary material for: HealthSWEDE: costs with sublingual immunotherapy—a Swedish questionnaire study
Source: Allergy Asthma Clin Immunol. 2021 Jun 7;17:55. doi: 10.1186/s13223-021-00560-3 (PMC8183062; doi:10.1186/s13223-021-00560-3)
Supplement: Supplementary file 2 — Additional file 2. Questionnaire. [file 13223_2021_560_MOESM2_ESM.docx]

**Additional file 2**

**HealthSWEDE: Costs with sublingual immunotherapy - a Swedish questionnaire study**

Petter Olsson, Carl Skröder, Lars Ahlbeck, Frida Hjalte, Karl-Olof Welin, Ulla Westin, Morgan Andersson, Cecilia Ahlström Emanuelsson, Lars-Olaf Cardell

ALLERGY: Health, health-care contacts and medication

**Part I: Background information**

**1** Gender: ❑ Female ❑ Male

**2** Year of birth? 19…….

**3** What is your present (main) work situation?

(You can select several alternatives)

❑ Employed/Self-employed fulltime

❑ Employed/Self-employed part time______________%

❑ Full time student

❑ Part time student___________%

❑ Other (e.g. unemployed, disability pension, long term sick leave, parent leave)___________________%

**4** **Do you smoke**?

(Please choose one alternative)

❑ Yes, Daily, more than 1 packet/day

❑ Yes, Daily, app. 1 packet/day

❑ No, Daily, less than 1 packet/day

❑ No, but I used to smoke every day

❑ No, I have never smoked

**5** Have you ever received one of following **diagnoses** set by our **Physician**?

(You can select several diagnoses)

❑ Allergic nasal and/or eye problems (including hay fever)

❑ Non-allergic nasal problems (“hypersensitive nose”)

❑ Asthma

❑ Skin rash

❑ COLD (Chronic Obstructive Lung Disease)

❑ No, none of the diagnoses above

**6** Do you have any **allergic symptoms** right now?

❑ Yes

❑ No

❑ Don’t know

**7** What are you **allergic to**?

(You can select several alternatives)

❑ Birch pollen

❑ Grass pollen

❑ Mite

❑ Dog

❑ Cat

❑ Bee/Wasp

❑ Other, …………………

**Part II: Allergic symptoms**

**8** Have you experienced discomfort because of allergic nasal- and/or eye problems during **the past 12 months**?

❑ Yes

❑ No

❑ Don’t know

**9** If you answered yes to question **8**: Did you experience discomfort because of your allergic symptoms **more than 4 weeks in a row**?

❑ Yes

❑ No

❑ Don’t know

**10** If you answered yes to question **8**: Did you experience discomfort because of your allergic symptoms **more than 4 days a week during the same week.**

❑ Yes

❑ No

❑ Don’t know

**11** During **how many months each year** do you experience allergic symptoms.

❑ less than 1 month

❑ 1-3 months

❑ 4-6 months

❑ 7-12 months

❑ I don´t experience allergic symptoms

**12** Have you ever experienced one the following statements despite of medical treatment/vaccination?

(You can select several alternatives)

❑ Sleep disorder

❑ Impaired ability in leisure and / or sports activities

❑ Impaired ability in school or professional activity

❑ Other disturbing symptoms

❑ No, none of the above

**13** Please grade your allergic symptoms from zero to ten. Zero is no allergic symptoms and ten is severe allergic symptoms.

Mark with a ring the grade which best suits your allergic symptoms.

**0**-----1-----2-----3-----4-----5-----6-----7-----8-----9-----**10**

**Del III: Use of medication and health-care contacts**

**14** Have you used medication because of allergic induced nasal/eye symptoms during the past year?

❑ Yes

❑ No

❑ Don’t know

**If your answer to question 14 was ”yes”, please answer question 15 – 21, otherwise continue to question 22.**

**15** Have you, **inspite use of allergy medication**, experienced discomfort related to your allergy, during the past 12 months?

❑ Yes

❑ No

❑ Don´t know

**16** During the past year, have you used any **nasal spray/powder containing cortisone** (e.g. Avamys^®^, Becotide Nasal^®^, Desonix ^®^, Dymista^®^, Flutide Nasal^®^, Nasonex^®^, Rhinocort^®^,) for your allergic nasal/eye problems?

❑ Yes, less than 1 month

❑ Yes, 1-3 months

❑ Yes, 4-6 months

❑ Yes, 7-12 months

❑ Yes, more than 12 months

❑ No

❑ Don’t know

**17** During the past year, have you used any **nasal spray containing antihistamine** (e.g. Lastin^®^ or Livostin^®^,) for your allergic nasal/eye problems?

❑ Yes, less than 1 month

❑ Yes, 1-3 months

❑ Yes, 4-6 months

❑ Yes, 7-12 months

❑ Yes, more than 12 months

❑ No

❑ Don’t know

**18** During the past year, have you used **decongestant nasal spray/drops** (e.g. Nasin^®^, Nasoferm^®^, Nezeril^®^ or Otrivin^®^) for your allergic nasal/eye problems?

❑ Yes, less than 1 month

❑ Yes, 1-3 months

❑ Yes, 4-6 months

❑ Yes, 7-12 months

❑ Yes, more than 12 months

❑ No

❑ Don’t know

**19** During the past year, have you used **antihistamine, tablets or liquid form**, (e.g. Aerius^®^ Clarityn^®^, Cetirizin^®^, Kestine^®^, Loratadin^®^, Telfast^®^ or Zyrlex^®^) for your allergic nasal/eye problems?

❑ Yes, less than 1 month

❑ Yes, 1-3 months

❑ Yes, 4-6 months

❑ Yes, 7-12 months

❑ Yes, more than 12 months

❑ No

❑ Don’t know

**20** During the past year, have you used **decongestant tablets** (e.g. Rinexin^®^ or Rinomar^®^) for your allergic nasal/eye problems?

❑ Yes, less than 1 month

❑ Yes, 1-3 months

❑ Yes, 4-6 months

❑ Yes, 7-12 months

❑ Yes, more than 12 months

❑ No

❑ Don’t know

**21** During the past year, have you been prescribed **cortisone tablets** (e.g. Betapred^®^, Prednisolon^®^) or **cortisone injections** (e.g. Depo-Medrol^®^) for your allergic nasal/eye problems?

❑ Yes, less than 1 month

❑ Yes, 1-3 months

❑ Yes, 4-6 months

❑ Yes, 7-12 months

❑ Yes, more than 12 months

❑ No

❑ Don’t know

**22** How many **times** during **the past year** have you been given **help** or **information** relating to your allergic nasal/eye problems from the following?

None 1 2-4 5-10 >10 Don’t know

Doctor’s visits ❑ ❑ ❑ ❑ ❑ ❑

Nurse’s visits ❑ ❑ ❑ ❑ ❑ ❑

Counselling by phone. ❑ ❑ ❑ ❑ ❑ ❑

Pharmacy staff at health

outlets or other ❑ ❑ ❑ ❑ ❑ ❑

alternative medicine

Internet ❑ ❑ ❑ ❑ ❑ ❑

❑ I have no your allergic nasal/eye problems?

**Del IV: Allergy vaccination**

**23** Have you received **”allergy vaccination”** as an injection during the past year (e.g. Alutard^®^) for your allergic nasal/eye problems?

❑ Yes, less than 1 month

❑ Yes, 1-3 months

❑ Yes, 4-6 months

❑ Yes, 7-12 months

❑ Yes, more than 12 months

❑ No

❑ Don’t know

**24** Have you been treated with **”allergy vaccination”** as **tablets** (Grazax^®^) for your allergic nasal/eye problems?

❑ Yes, my vaccination is ongoing

❑ yes, my vaccination treatment is finished

❑ No

❑ Don’t know

**If your answer to question 24 was ”Yes”, please answer questions 25-27**

**25 If your answer to question 26 was ”ongoing”**; during how many months have you received vaccination treatment in total?

❑ less than 1 month

❑ 1-3 months

❑ 4-6 months

❑ 7-12 months

❑ more than 12 months

**26 If your answer to question 26 was ”Finished”**; since when was your vaccination treatment finished?

❑ less than 1 month

❑ 1-3 months

❑ 4-6 months

❑ 7-12 months

❑ more than 12 months

**27** During your allergy vaccination, have you ever experienced an **“allergic chock”**?

❑ Yes

❑ No

❑ Don’t know

**Del V: Sick leave from work or studies because of allergic nasal/eye problems**

**28** How many days during the **past year** have you been absent from work/studies because of allergic nasal/eye problems?

0 days 1-7 days 8-14 days 15-30 days other

❑ ❑ ❑ ❑ ❑_________

**29** Can you estimate how many days in total during the **past year** you had allergic nasal/eye problems and **still went to work/studied**?

0 days 1-7 days 8-14 days 15-30 days other

❑ ❑ ❑ ❑ ❑_________

**30** If you think about the days in Question 29, how would you describe your average **performance** at work/­studies?

Put a cross on the line.

Fully active Operated at half speed Completely absent

100% 75% 50% 25% 0%

**31** Please answer the questions in the quality of life survey EQ-5D as well, where you estimate your health today.

Thank you for your participation!

Ear-, nose- and throat Department

Skåne University Hospital, Lund

Contact/questions:

Phone number:
